# “VR is the future”: perspectives of healthcare professionals on virtual reality as a diagnostic tool for dementia status in primary care

**DOI:** 10.1186/s12911-023-02413-y

**Published:** 2024-01-04

**Authors:** Joshua Yondjo, Joyce Siette

**Affiliations:** The MARCS Institute for Brain, Behaviour and Development, Western Sydney University, Westmead, NSW 2145 Australia

**Keywords:** Dementia, Virtual reality, Healthcare professionals, Primary care

## Abstract

**Background:**

Healthcare professionals (HPs) hold critical perspectives on the barriers and facilitating factors for the implementation of virtual reality (VR) dementia diagnosis tools in the clinical setting. This study aims to explore HP perspectives regarding the clinical implementation of dementia diagnosis tools using VR platforms.

**Methods:**

An exploratory qualitative interview study was carried out between July and September 2022. In-depth semi-structured interviews were conducted with HPs (n = 7) with clinical expertise in dementia diagnoses drawn from medicine, nursing and allied health practices. A hermeneutic phenomenological approach was used to frame the interview data across the dementia diagnosis pathway and application of new technology.

**Results:**

HPs were on average 36.29 years old (SD = 11.56) with 11.85 years of experience (SD = 12.80, range:4–42). Analyses identified three main themes related to the contemporary methods of dementia diagnosis, dementia diagnosis and the medical landscape and HP perspectives on the usefulness and barriers of VR implementation. VR was considered an innovative prospect, with improved ecological validity compared to commonplace, current cognitive assessments. Concerns of time commitments, monetary costs and the validity of the new technology were identified as key barriers to implementation. Overall, implementation of a new diagnostic tool was considered a complex process.

**Conclusions:**

Our insight into general practice and nursing clinics can be supported to embed and integrate virtual reality platforms in primary care settings. Primary healthcare organizations require more funding and time related resources to produce a context in which VR tools could be implemented in a beneficial manner.

**Supplementary Information:**

The online version contains supplementary material available at 10.1186/s12911-023-02413-y.

## Introduction

Dementia is a neurodegenerative disorder, commonly associated with later stages of ageing, that results in the gradual atrophy of cognitive ability [1]. Projected increases to individuals living with dementia are expected to reach 152 million by 2048 [2], causing substantial strain and burden on worldwide communities and healthcare systems. Early detection can prevent costly treatment for the healthcare system while allowing for preventative measures to be established [3].

Dementia diagnosis is a multifaceted challenge within healthcare systems and often requires a comprehensive understanding of patient data achieved through collaborative efforts among healthcare professionals [4]. In particular, the roles played by general practitioners (GPs) and primary care nurses in this diagnostic process are key, given their frontline positions in patient care. GPs, serving as primary care physicians, often function as the initial point of contact for individuals exhibiting cognitive concerns [5, 6] Their responsibilities encompass conducting preliminary assessments, facilitating specialised referrals (e.g., for neuropsychological testing), and collaborating with other healthcare stakeholders (e.g., geriatrians) to establish a comprehensive diagnostic framework [7, 8]. Simultaneously, primary care nurses assume a critical role in the ongoing care and monitoring of individuals diagnosed with dementia, offering unique insights into patients’ daily lives, behavioural changes, and responses to interventions [9–11].

Current contributions of GPs and nurses in the diagnostic landscape highlights the need for improved dementia diagnosis tools [12–14]. Current measures, including widely used assessments like the Mini-Mental State Examination (MMSE) [15] and the Montreal Cognitive Assessment (MoCA) [16], exhibit limitations in assessing temporal and visuospatial changes in cognitive domains, particularly within the often unrealistic and stressful setting of primary care [17]. Notably, executive function, perceptual motor function and social cognition, key indicators of dementia progression [18], remain unaddressed in existing diagnostic tools [17]. Compounded by practical issues such as cost and time constraints in administering these tools, current diagnostic methods faces significant challenges [19]. These barriers may impede the timely delivery of diagnoses at critical timepoints of individuals, necessitating the exploration of alternative measures.

Virtual reality (VR) allows for an immersive and intrinsically motivating user experience through computer generated environments [20]. Over the last two decades, advancements in producing cost-effective yet powerful computer hardware has significantly increased the perceived viability of VR among healthcare professionals for different uses [21, 22]. In the realm of dementia diagnosis, non-immersive alternatives, such as the RE@CH assessment which uses motion sensor technology [23], are employed to gauge the simulation of cognitive performance in older adults during simulated everyday tasks. These tasks vary from numerical input for door passcodes to the identification of commonplace objects and navigation within spatial contexts [23]. A recent review has highlighted the potential utility of diverse immersive VR assessment conditions, encompassing simulations and structured evaluations of cognitive performance, in discerning cognitive impairment [24]. Despite the developing literature on VR tool efficacy, most GPs have concerns on the practicality of using VR more generally for older patients [25]. VR has been perceived by GPs as confusing for older adults to navigate, with a need for prior practice before cognitive assessments can be accurately administered [25]. Additionally, GPs suspect age-related degradation in hearing and vision may limit performance or facilitate nausea related side effects like headaches and dizziness. Yet, multiple studies suggest older patients pick up novel VR technology quickly and with minimal assistance [21, 26]. Whilst VR tools may be a promising avenue for cognitive diagnosis, GPs and nurses perceptions of VR diagnostic tools need to be fully considered to support its use and implementation.

Our study thus aims to investigate GP and nurses perspectives surrounding the accessibility and operation of VR diagnostic technology, including anticipated barriers to efficacy and application. Findings will add to the established literature and inform the development of future VR diagnostic tools for cognitive impairment.

## Methods

### Design

Individual semi-structured interviews were conducted with GPs and geriatric nurses situated across the greater Sydney area between July and September 2022. This study was approved by the Western Sydney University Human Research Ethics Committee (H14896).

### Recruitment and sampling

A purposive sampling method was used to recruit GPs and primary care nurses by both study authors. This involved distributing flyers advertising the study to physical locations (e.g., general practices, memory clinics), e-newsletters sent via researcher networks and disseminated to national not-for-profit organisations (e.g., Australian Association of Gerontology). Emails were also sent to general practices across New South Wales, Australia with either the study flyer or information regarding the study.

### Participants

Clinically registered healthcare professionals were eligible for participation in this study. The inclusion criteria targeted healthcare professionals, specifically general practitioners (GPs) and primary care nurses, who possessed a minimum of two years of experience in the field of primary care. Additionally, preference was given to GPs with specialisation or substantial experience in geriatrics or neurology. Participants were required to be actively practicing in primary care settings, including general practices, clinics, or community health centers within the geographical region of New South Wales, Australia. This choice of participants based on these criteria aimed to ensure a targeted and knowledgeable cohort with direct involvement in dementia diagnosis within the local healthcare context. Through our recruitment procedure, one geriatric nurse, and six practicing GPs contacted the research team, received written study information and provided informed written and verbal consent. Participants were aged between 29 and 64 years (M_age_=36.29, SD = 11.56) and had on average 11.85 years of experience (SD = 12.80, range:4–42).

### Data collection

Semi-structured interviews with participants took place either in person or over the video conferencing software, Zoom. Before interviews commenced, participants were briefed on the study and provided a participant information sheet. The interview commenced once informed verbal and written consent was obtained and the participant had been briefed.

**Fig. 1:**
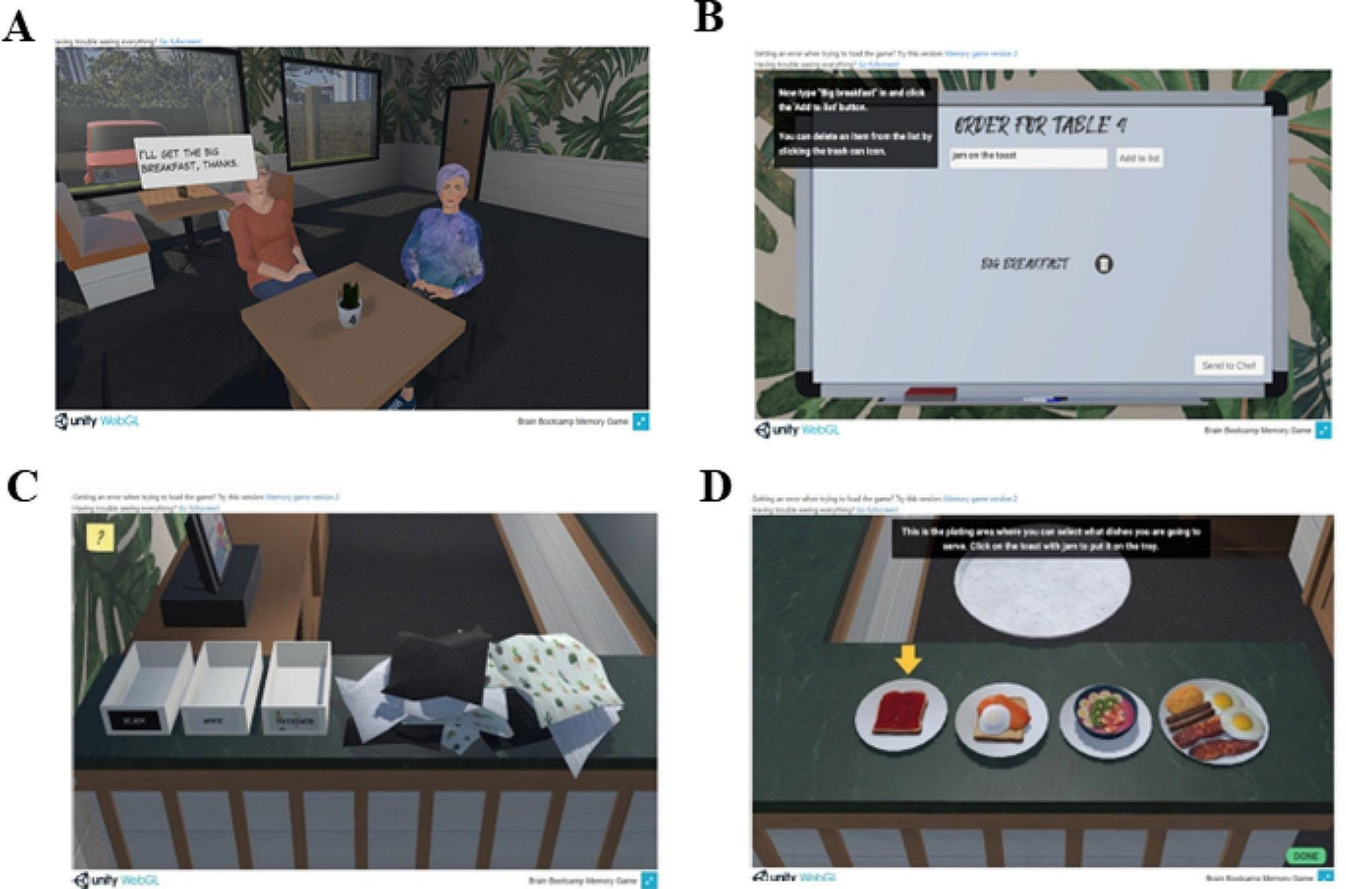
Screenshots of the virtual reality platform (LEAF CAFÉ) developed by the research team to assess for cognitive impairment. The platform uses a café setting to ask patients to obtain a food order (**A**), record the order (**B**), complete sorting activities (**C**) and collect the order (**D**)

The interview schedule was sourced from prior research [22, 27, 28] with minor alternations to suit the purpose of this study and focused on general questions regarding their experiences of diagnosing dementia (e.g., “How do you currently diagnose a patient with cognitive impairment or dementia?”), current resources (e.g., “How would you improve or change about the current information you can access?”), before being shown screenshots of the research team’s sample VR dementia diagnosis module (see Fig. 1) as an exemplar of a clinical application of VR (“Do you think this form of technology would be considered useful in a primary care setting?”). Development of this computer simulated environment to screen for cognition is described elsewhere [29]. Briefly, the virtual restaurant module, developed with the Unity game engine and accessible through web browsers, immerses users in a café scenario where they act as waiters serving customers. Simulating real-world activities, the module assesses various cognitive functions, including processing speed, learning and memory, and executive functioning. Users perform tasks such as taking and recalling customer orders, navigating distractions, and delivering dishes to the correct tables. The module consists of five levels, each increasing the memory load, and users receive a score based on their performance in completing tasks accurately. The scoring algorithm evaluates immediate and delayed recall of orders, selection of correct meals, and spatial memory in serving dishes, providing a comprehensive assessment of participants’ cognitive abilities in a virtual restaurant setting.

Following a brief introduction of the module, participants were asked to share their perspectives on this technology form, its clinical application (e.g., usefulness of tool in making diagnostic decisions) and future barriers (e.g., “What are the potential impoementation barriers of this new technology for you?”). Interviews were conducted by JY (who received training from JS), then recorded and transcribed verbatim using the online tool Otter.ai and the Descript app with some manual transcription being required. No other field notes were collected. After data collection concluded, each participant was sent a $20 gift voucher. Interviews continued until data saturation was achieved, at which no new information or themes emerged from successive interviews. During the transcription process participant names, location and other identifiable information was removed. The interview guide is available in Supplementary File Table S1.

### Data analysis

A hermeneutic phenomenological approach was used, which assumes that individuals perceive an objective reality and derive a subjective lived experience from their perception [30].

Braun and Clarke’s [31] six step approach to thematic analysis was applied to the inductive thematic approach. Inductive category development in qualitative research aims to facilitate the organic emergence of themes and patterns from data, allowing researchers to uncover unexpected phenomena and insights, especially in the context of exploring novel or under-researched topics [32]. This approach prioritises a participant-centric analysis, emphasising understanding from the participants’ perspectives rather than imposing pre-existing theoretical frameworks [32]. By doing so, findings are grounded in the authentic experiences of participants, contributing to a richer and more detailed description of the phenomenon under investigation [33]. Indeed, our aim was to understand how healthcare professionals considered their role in dementia diagnosis within the primary care setting and explored perspectives which had been shaped by interpretations of their own lived experience.

Initial independent reading and re-reading of five transcripts were performed by two researchers (JS and JY). The researchers then collaboratively formulated a coding structure during their meeting, which marked the first step. Subsequently, significant data points were initially coded in step two. Step three involved comparing codes from all participants and refining them into categories based on content similarity, leading to the development of initial subthemes. In step four, the initial subthemes were reconstructed and condensed into subthemes that more accurately represented the interview data. The identification of major themes was achieved by refining these subthemes, and each major theme was succinctly named. If themes could not be resolved then a third researcher (JM) was consulted. For example, initial subthemes were derived from participants’ discussions on diagnostic methods, including the use and limitations of traditional assessment tools, as well as challenges and nuances in the diagnostic process (e.g., multifactorial nature of dementia diagnosis, the significance of patient history, and the specific challenges), which helped to form the major theme of *Contemporary Methods of Dementia Diagnosis*.

## Results

**Fig. 2:**
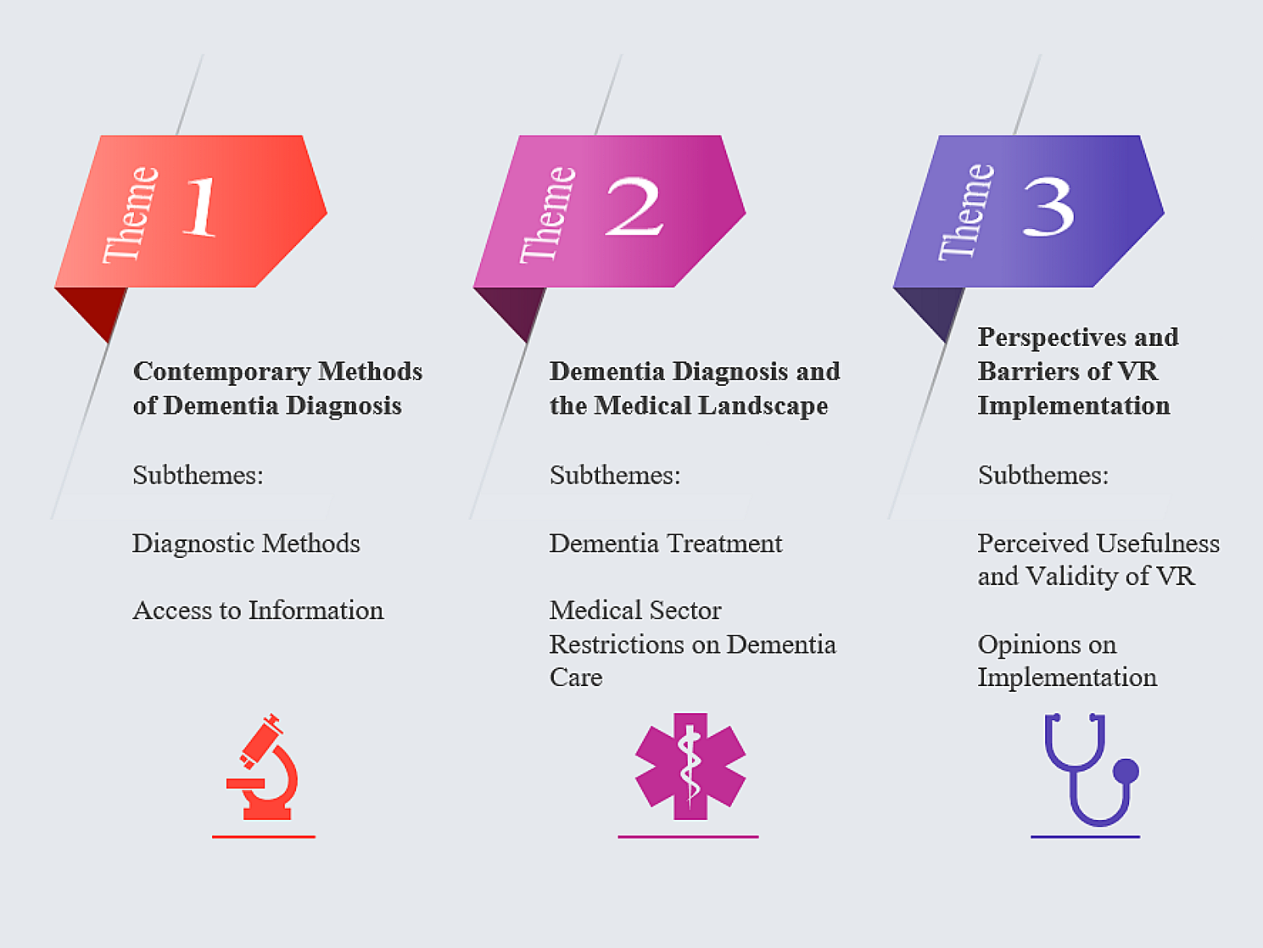
Summary of main themes and sub-themes

The thematic analysis produced three main themes and six sub-themes (see Fig. 2).

### Contemporary methods of Dementia diagnosis

This theme explored current methods and processes employed by GPs to produce a dementia diagnosis. This included examination of the main methods of investigation and assessment as well as the processes of how general and patient information are sought for diagnosis.

#### Diagnostic methods

All participants described dementia diagnosis as a multifactorial, lengthy process, and focused on factors such as a patient’s physiological symptoms, their cognitive constitution and their biographical and social history.

History examination, and we may use the RUDAS or MMSE or the MoCA. And obviously there are other things like blood tests and imaging, CTs to brains, MRIs, etc. … you can do it again six months, a year, whatever down the track, see if there have been changes. [GP1]

While traditional tools such as the MMSE or MoCA are useful in detecting moderate cognitive impairment, GPs often regarded them as blunt tools, unable to detect subtle signs of early dementia. GPs also supported neuropsychological assessments for their sensitivity to early dementia symptoms. However, access to these tools was limited by long wait times and high assessment costs. These objective tools also lack ecological markers.

[Patient]… often misplaces things or leaves the stove on, none of that’s actually in the mini mental. So there are ways in which someone might be impacted that’s not in an objective scoring system. [GP2]

The diagnosis process mostly explored a patient’s history, which can take up to 70% of the investigation and covered cognitive symptoms (e.g., memory, language skills and judgment), behavioural changes (e.g., sleeping problems, restlessness), psychiatric symptoms (e.g., depression and anxiety, personality changes) and neurological symptoms (e.g., abnormal gait or lapses in vision). GPs found that investigation into these areas could aid in detecting gradual or sudden declines in cognitive ability. Despite its usefulness, the process of gathering history can be lengthy.

I usually book a double or a triple appointment [as] it can’t be done in a single appointment. [GP3]

A patient’s corroborative history was instrumental for initiating dementia investigations, with more emphasis placed on history originating from the medical expertise of geriatric nurses or from observation and prior assessment data.

Sometimes people themselves are not aware that their cognition is failing. And they insist that there is nothing wrong with them and so it’s hard then to get information from them if they’re denying that there’s a problem. [GP1]

Within the Indigenous population, dementia symptoms are commonly considered as signs of ageing rather than pathology. In these communities, differences in understandings of health, and different belief systems often resulted in poorer dementia outcomes compared to urban areas. Issues in making treatment accessible to these communities were also discussed as culturally appropriate tools remain difficult to identify. These barriers required practitioners to be culturally competent in order to understand and treat Indigenous patients.

It can be quite difficult for Indigenous patients that have a low level of health literacy also being able to not just understand the diagnosis and the information pertaining to things like management, but also navigating health services. [GP6]

#### Access to relevant information

Dementia diagnosis required the elimination of extraneous causes (e.g., infection induced delirium) through reading and sourcing additional, accessible information and guidelines from outlets such as Mayo Clinic, The Centers for Disease Control and Prevention and Google.

The Royal Australian College of General Practitioners (RACGP) has some good articles on dementia and I usually go for Up to Date as well. I really like Up to Date, which is kind of like a, a doctor’s Google. [GP3]

The GPs interviewed described areas concerning general and patient information that they think could be improved. Providing GPs with a clear and relevant assisting history would be helpful in constructing a comprehensive bio-psychosocial profile for patients.

### Dementia diagnosis and the medical landscape

This theme specified the ways in which the medical institution impinges on the way GPs diagnose dementia. The subthemes examined the treatment options available to GPs and areas lacking in the medical institution that cause strain on the quality of dementia care.

#### Dementia treatment

Specialists are an imperative aspect of early dementia detection. Neuropsychologists are apt in detecting early dementia symptoms, allowing for the swift institution of counter measures. All GPs interviewed noted a reliance on access to geriatricians for complex cases and formal diagnosis.

If I have any concerns about my decision making and it’s not clear, then I’ll speak to one of the geriatricians. [GP5]

GPs expressed that a geriatrician’s expertise resulted in patients trusting them more than GPs, thus allowing geriatricians to set standards for cognitive assessment protocol. This extends to the acceptance of novel tools and assessments.

Most clients won’t want to do something that’s quite novel, if it was really well established and linked to it like a geriatric clinic, by all means you know, patients would think I’m getting that quality care, and at the forefront of technology but if a GP were to offer it they would just be like ‘what are you doing? [GP2]

#### Imposed restrictions

A major barrier to the diagnosis process mentioned by all GPs interviewed was the lack of time available in daily practice. Current general practice restrictions force GPs to improve their efficiency to suit consultation demand. GPs noted the need for empathetic and thorough practice, despite this emphasis on efficiency. Furthermore, time constraints as a key barrier extended past general practice and contributed to the limited access to geriatricians that GPs experienced.

It’s hard to get a good a neuropsychological screening done, because it takes two or three hours. It has to be done through hospital outpatient where it’s a very long waiting time or through a private psychologist where it costs several hundreds of dollars or more which a lot of people can’t afford. And getting access to geriatricians is again, not that easy because most of them are based in hospital clinics and again there is waiting times. [GP1]

Many older patients cannot afford the care they need. Despite many older patients not being able to afford treatment, the treatment prices put forth by the medical institution remain high. GPs suggested this worsening problem could be countered by increased funding to the community, nursing home and hospital sectors. This funding could also improve medical infrastructure, as technology used in general practices can be ten to twenty years old.

### Perspectives on the usefulness and barriers of VR implementation

This theme focused on participant perspectives towards the implementation of VR in the clinical setting, including its perceived usefulness, the validity of current diagnostic tools and facilitating factors supporting implementation.

#### Perceived usefulness and validity of VR

Most participants gave some positive appraisal to the usefulness of VR in the clinical setting and considered this format to be a relatively easy and approachable means of assessment.

Because you’re using a lot of visual cues, which is something that a lot of current sort of scoring systems and tests don’t do so much and it’s interactive as well. So it’s, it’s a really sort of dynamic and different way of approaching an age old problem. [GP2]

The ability to immerse patients in a variety of virtual environments without the need for physical equipment was an aspect participants considered convenient for general practice and nursing settings.

VR is more of a game type thing. Some people might find it less threatening and easier to go through than answering direct questions. [GP1]

GPs suggested that depending on the practice population, VR implementation could come with real practical benefits in dementia diagnosis.

I would use it at least two or three times a week, because I have a population that’s largely elderly. [GP1]

However, participants outlined multiple stages that need to be thoroughly developed prior to the clinical use of VR technology to ensure VR assessment is cheap, simple and time effective. GPs questioned the practicality of VR tools in general practice, as effects of ageing like vision loss could make VR assessments difficult for patients to navigate. For general practices, the prospective costs of VR implementation raised concerns on whether investing in VR is worth the access to something that may not bring major change to quality or quantity of life for dementia patients. Thus, VR assessment may be better suited for specialist clinics with government funding.

Early detection’s main purpose is so that family members can be aware and then maybe value the time that they have left with their loved ones. [GP2]

VR tools were seen as having a strong theoretical background by healthcare professionals. However, participants drew differing intuitions on the areas of cognition the theoretical background would apply to. For instance, some participants expected VR tools to be limited to measures of memory retention, similar to currently used paper and pencil tools. Other participants suggested areas of executive functioning that apply to spatial reasoning could be examined through VR assessment.

The most common concern brought up by participants was the validation of VR assessments and its performance across different psychometric properties. High ecological validity was an aspect of VR assessment perceived by participants to be beneficial to dementia diagnosis. Virtually reproducing the tests required for diagnosis could bring new possibilities for patient history investigation, resulting in a more streamline and detailed process.

The idea of putting a patient into a safe, but kind of imitating real life situation to practically assess their levels of dementia, that’s really novel and I think that that has really amazing future implications for diagnosis and assessment of dementia in a way that we just currently cannot assess it. [GP3]

Some participants suggested that there may be limits to the ecological validity of VR tools. Participants found VR to lack the propensity to perfectly emulate all aspects of sensory experience (e.g., sensitivity to touch, vision, hearing, balance). These shortfalls of VR technology raised concerns on whether virtual tasks and the resulting measurements would be comparable to their real life counterparts. VR assessment were also found by participants to lack the complexity of human interaction which can elucidate deficits in communication and social awareness.

Even just to see how they react. In the way they choose their words and whether they’re struggling to find their word which most people wouldn’t struggle with. [GP1]

Older patients’ ability to understand and use VR tools was a potential barrier for future implementation. Despite the possible benefits, technological literacy was considered by HPs to be low in older populations and less suitable for VR assessment, since assessments may reflect their ability to use VR technology more than their cognitive ability. Patients unfamiliar with VR may further feel belittled by the use of a game like assessment.

There’s always individual elements like some people just don’t like doing this kind of tests because they feel, ‘Do you think I’m stupid?’ those sorts of things. [GP1]

Societal shifts towards technology was another factor that made VR assessments appealing to participants. However, adoption could be slower in Indigenous communities due to financial and geographic barriers.

The low socioeconomic areas, where many Indigenous peoples reside, access those technologies after everyone else. [GP6]

Once implemented VR tools were considered more useful as they are more accessible, however its use is contingent on how well practitioners can integrate VR with the current system.

#### Opinions on implementation

Participants had differing opinions on where and how VR tools would best be implemented. Most participants interviewed preferred nurse clinics and patients’ homes as places for VR implementation, as these contexts require smaller financial and time investments from GPs. A small number of GPs preferred general practice implementation due to superior quality control in test conditions when cognitive tests are being administered by GPs.

VR assessments can be completed at home via the Internet. [GP2]

A clinical setting can account for differences in computer literacy among patients. [GP1]

VR tools may be better suited for nurse clinics as a neuropsychological assessment. [GP3]

Participants desired the results from VR assessments to be presented as scores similar to MMSE or MoCA scores, with a short summary report including a conclusive statement. This would be most useful if made available on the Medical Director or Best Practices software. Results should also be accompanied by references that explain the meaning of each measure and scoring range. These references would aid nurses and other HPs in further understand a patient’s symptoms.

Another barrier to the adoption of VR tools in primary care involved a lack of financial remuneration for GPs and practices. It was voiced that even if VR assessments were remunerated through Medicare, the return was anticipated to be low given current geriatric assessments are poorly funded. VR financial feasibility was a barrier that could also extend to nursing regardless of its clinical benefit.

You don’t get remunerated for it, like there is no Medicare item for it. So that’s 15 min doing a cognitive assessment to VR for this patient, and a lot of things end up going back to billing. [GP2]

Time constraints were another major barrier to practice and GP adoption of VR assessments. This issue was compounded by the perception that VR tools may not produce major changes in the subsequent decisions GPs make compared to current tools.

From a timing perspective, I don’t see how practically a lot of GP practices would have the time to be doing this formal assessment versus where we would, and I’m unsure how practically this would be better than our standard assessments that we’re currently doing. [GP3]

## Discussion

This study explored the perspectives held by healthcare professionals (HPs) on the implementation of VR diagnostic tools for cognitive impairment. During this investigation, participants elaborated on current dementia diagnostic practices and how VR tools could augment this process. Participants expressed constraints on the time they have in practice, the amount of dementia related funding available and their access to more advanced forms of dementia diagnosis and treatment. This past experience directly informed their appraisals of VR tools and their implementation. All participants regarded VR assessment as a promising prospect, with potential gains in dementia care and outcomes, however time and cost effectiveness were also key considerations.

Most HPs interviewed found the proposed applications of VR tools to be easy and approachable for both HPs and patients. Clay et al. [24] found corresponding responses from participants in their study, with reports that immersive VR assessments were an enjoyable experience which may give a therapeutic advantage to VR tools. The advantage of VR extends to the ability to virtually replicate various real life conditions and cognitive decline specific assessments in a virtual space [23, 24]. HPs from this study described this prospect as a rich source of innovation for future cognitive screening methods.

A major concern mentioned by all participants was the validity of VR tool measures, with apprehensions on the ability for VR assessments to accurately measure dementia and represent real life conditions. However, recent advances suggests that VR tools are significantly consistent with standardised cognitive screeners such as the MMSE and MoCA [6]. Furthermore, immersive VR platforms can also utilises the measurement of kinematic movement as a means of portraying real life conditions [34] to support ecological assessments. Nevertheless participants voiced concern on whether the measures from VR tools were genuinely sensitive to dementia cases. To investigate this, applying VR tools to cases of dementia with differing severities would produce a standardized measuring scale and potential customisable VR tools which could adapt to patients on a case by case basis. However, consideration of how customisable tools may compromise validity is required.

Time taken to learn how to use VR tools and the possible administration time required for effective diagnosis was also a major concern in this study and prior research [35]. Whilst Bayahya et al. [36] suggested that VR would alleviate both time constraints experienced by clinicians and high costs associated with diagnosis, this may take time to action. Indeed, HPs voiced concerns regarding new implementation process could result in more time and money being committed compared to current practices initially. Prior research echoes this finding that after administering a trial using VR exercise therapy, physical therapists found the time required for set up and maintenance of VR tools to be a barrier [35]. However, these previous concerns may be due to clinicians being inexperienced with VR technology, rather than VR technology itself being onerous and inefficient [37]. Future research needs to ensure that VR platforms remain service-friendly and compatible with existing practices and systems to ensure optimal adoption. Providing training with the intended users, as well as having a champion user, could also be beneficial.

General practices may not be the only complementary platform for VR implementation. Our results suggest that HPs’ sentiment towards VR implementation ranges across multiple contexts at a wider scale (from home-based self-initiated processes to nurse-led assessments at clinics), with most HPs preferring VR to be used outside of general practices. Indeed, completing a VR assessment at home can provide a familiar environment as opposed to a clinical setting, and can soothe patient test anxiety. This is also a feasible approach which could help reduce time constraints experienced by HPs, with recent findings suggesting that patients could competently administer VR tasks themselves with results being saved to cloud-storage [38].

Overall, major concerns towards VR included financial costs, administration time, tool validity and next steps. Providing access to accessible, functional and reliable tools with focused guidelines that outline the diagnostic features and pathways of particular types of dementia is suggested. Such tools are likely to be accepted and adopted in the healthcare system.

### Limitations

This study had several limitations. Firstly, the disproportionate distribution of GPs and geriatric nurses raises questions about the transferability and generalisability of conclusions, particularly for individual occupational groups. Whilst similar themes were found in this group, the small sample size as well as the restricted geographic focus, encompassing only health professionals in the greater Sydney and rural New South Wales areas, further diminishes the study’s potential for broader applicability. Furthermore, the limited exposure of participants to the VR tool during the introductory phase, without the opportunity for firsthand experience, poses a significant constraint, potentially impacting the depth of insights into the utility and limitations of VR dementia diagnosis tools. Recommendations for future research include widening the participant pool to include diverse occupational groups and presenting a validated VR tool to more diverse healthcare professionals, including geriatricians and neuropsychologists, before the semi-structured interviews to capture a more comprehensive perspective on these emerging technologies and procedures.

## Conclusion

VR dementia diagnosis tools appear to be a promising avenue of technological development in primary care, however in its current state it may not be necessarily required in general practice. As both older patients and GPs are resource poor, the investments required to adopt VR tools is a major barrier. Despite this, if validity for these tools is established and costs to patients and practices are addressed, VR technology may become an asset to the dementia diagnosis process.

### Electronic supplementary material

Below is the link to the electronic supplementary material.

Supplementary Material 1

## Data Availability

The datasets generated and/or analysed during the current study are not publicly available due to identifiable information in the interviews but are available as composite data from the corresponding author on reasonable request.
