# Supplementary material for: “VR is the future”: perspectives of healthcare professionals on virtual reality as a diagnostic tool for dementia status in primary care
Source: BMC Med Inform Decis Mak. 2024 Jan 4;24:9. doi: 10.1186/s12911-023-02413-y (PMC10765843; doi:10.1186/s12911-023-02413-y)
Supplement: Supplementary file 1 — Supplementary Material 1 [file 12911_2023_2413_MOESM1_ESM.docx]

**Supplementary Files**

**Table S1.** Interview Schedule

| No. | Question |
| --- | --- |
| 1 | How do you currently diagnose a patient with cognitive impairment or dementia? |
| 2 | What clinical information is most important to make clinical decisions for clients at risk of cognitive decline? Why? |
| 3 | Where do you currently access information to make your diagnostic decision regarding dementia status? |
| 4 | What further information do you not currently have, but wish you had access to? Why? |
| 5 | What would you improve/change about the current information you can access? |
| 6 | Have you experienced difficulties accessing information previously? Why was it difficult? |
| 7 | What information is easier or harder to access? |
| 8 | What are your initial opinions using VR games? |
| 9 | Do you think VR can better serve the diagnostic decision-making process compared standard practice? |
| 10 | Do you think this form of technology would be considered useful in a primary care setting? How/where would you like this game utilised? |
| 11 | How would you like the information from the game presented to you? What about to your patients? |
| 12 | What would an ideal presentation look like for you? |
| 13 | How would you ideally like to be able to use the data from the VR game? |
| 14 | Which group of clients do you think would benefit most from this mode of screening? |
| 15 | How would you like to learn how to use the platform presenting the data? |
| 16 | Is the game necessary/useful for making diagnostic decisions or do other tools provide similar information? |
| 17 | What are the potential implementation barriers of this new technology for you, your practice, and clients? |
| 18 | How can this form of technology be smoothly implemented into a clinical setting? |
| 19 | Any other comments/suggestions? |
